# Development and Validation of Photo‐Numerical Scales for Facial Wrinkles on Chinese Population

**DOI:** 10.1111/jocd.70451

**Published:** 2025-09-18

**Authors:** Xiaomin Zhao, Yang Yang, Yan Zhong, Hao Ouyang, Claude Saliou, Lily I. Jiang

**Affiliations:** ^1^ China Innovation Labs Estee Lauder Companies Innovation R&D (China) Co., Ltd Shanghai People's Republic of China; ^2^ SGS CSTC Standards Technical Services Shanghai People's Republic of China; ^3^ The Estée Lauder Companies Melville New York USA; ^4^ SGS Testing & Control Services Singapore Pte Ltd Singapore Singapore

## Abstract

**Background:**

Various ethnicities age differently due to intrinsic genetic factors and inherent responses to UV exposure. The unique facial skeletal structure and function of Chinese skin can result in distinct aging signs compared to those observed in Caucasians. Additionally, there are limited published photo‐numerical scales for cosmetic treatments, which often yield relatively low efficacy compared with aesthetic surgery. Therefore, appropriate and sensitive clinical evaluation tools are needed to accurately assess signs of aging and the efficacy of cosmetic treatments for the Chinese population.

**Aims:**

Develop a new set of photo‐numerical scales to evaluate facial wrinkles specifically based on a Chinese population.

**Patients/Methods:**

Four photo‐numerical wrinkle scales were developed based on Visia‐CR images from 5310 Chinese females aged 18–69, focusing on forehead, glabella, crow's feet, and nasolabial fold wrinkles. These 10‐point (0–9) scales were rigorously validated for repeatability, reliability, and usability through inter‐ and intra‐grader assessments conducted by multiple clinical research labs. Additionally, the correlation between these scales and objective 3D measurements from the Primos 3D instrument was analyzed. It was further assessed whether the scales were more appropriate and relevant in assessing Chinese skin wrinkles severity when compared to Caucasian skin or mixed population skin scales.

**Results:**

Forehead, glabellar, crow's feet, and nasolabial fold wrinkle scales were established based on the Griffith principle with a 10‐point harmonious scale. Inter‐ and intra‐grader validation data among 5 graders from different regions in China showed very high correlation scores, with ICC > 0.9 for intra‐grader and ICC > 0.8 for inter‐grader validation. The results indicate that the scales are highly reproducible and reliable when used by trained graders. Moreover, the grading results by naïve graders correlated well with those by expert graders, suggesting that the scales are user‐friendly and easy to apply. A comparison between grading scores and Primos data revealed a strong correlation between wrinkles' volume and grading scores (*r* = 0.79) and between wrinkles' depth and grading scores (*r* = 0.82). This crucial validation confirmed that the core range of the scales (mild to moderate, the general target population for cosmetic products) is particularly effective. When compared to previously used Caucasian skin scales, the grading scores obtained from the Chinese wrinkle scales showed significantly higher mean wrinkle severity values (*p* < 0.001) for the same set of photos, indicating that the new scales are scientifically sound, practical, and suitable for a Chinese population.

**Conclusions:**

In conclusion, the photo‐numeric wrinkle scales have demonstrated strong repeatability and reproducibility, high practicality, and reliable correlation with objective measurement. Given the tremendous sample size covering various regions in China, these scales are beneficial tools to evaluate the effectiveness of cosmetic products in a Chinese population.

## 1 Introduction

Skin aging is a multifactorial process influenced by both genetic and exposomal factors. Various ethnicities age differently due to intrinsic genetic factors and inherent responses to UV exposure. Genetically determined skin type is one of the main influencing factors; darker skin contains more melanin, a natural UV protective polymer embedded in the skin that helps protect the skin from UV damage [1, 2, 3, 4]. Facial structure differences are another main genetic influencing factor. Characteristics for Asians can include a weaker skeletal framework, a wider intercanthal distance, and a broad prominent forehead [4]. There are also differences in skin function among different ethnicities. For example, studies have reported that Asian skin may have a higher lipid content in the stratum corneum and stronger barrier function compared to Caucasian skin, which may lead to the delayed appearance of aging signs [2]. These distinguishing anatomical features suggest that signs of aging may present differently in Asians compared to Caucasians, especially with respect to facial ptosis and the location of facial wrinkle formation.

Sun exposure and pollution are two key exposomal factors leading to skin aging. These extrinsic factors in combination with varying intrinsic factors result in differential aging progression and signs of aging in different ethnicities. Although higher melanin content in the skin may provide some protection to the skin from UV damage, increased melanin can also lead to hyperpigmentation, which is typically perceived as a sign of aging.

Given the differences in skin structure and function across ethnicities, it is not surprising that the occurrence of facial aging signs and their progression also can differ [5]. Consequently, appropriate and sensitive clinical evaluation tools are needed to assess signs of aging and cosmetic treatment efficacy in specific ethnicities with ethnicity‐relevant photo‐numeric scales.

We set out to develop a set of photo‐numeric scales to evaluate one of the most important aging signs, facial wrinkles. Four different regions of facial wrinkles (forehead, glabella, crow's feet, and nasolabial region) were assessed specifically on a population of Chinese subjects. In this article, we describe the method for developing the photo‐numeric scales, and its validation, usability, and application in comparison to similar scales conducted on a Caucasian or mixed population.

## 2 Material and Methods

Photos: The SGS photo database collected over 2.5 years of imaging with 5310 Chinese females between 18 and 69 years old from January 2021 to August 2023, and age distribution was listed in Table 1. All the images were captured using VISIA‐CR Gen2 (Canfield Scientific, Fairfield, NJ) as the basis to establish new photo‐numerical scales including facial wrinkles (crow's feet, glabellar, forehead and nasolabial fold), and the same model of Visia‐CR was intended to ensure the lighting condition was as comparable as possible. Images taken under standard 1 lighting mode were used for all scales; and front and profile views were collected to reflect multi‐angle skin aging signs. All clinical studies involved to collect photos were conducted following Good Clinical Practice guidelines and China's regulations. Informed consents and photo release forms were obtained from all participants.

**TABLE 1:** Age distribution of the 5310 photo database.

Age	Number	%
< 20	40	0.75
20–29	353	6.65
30–39	1120	21.09
40–49	1758	33.11
50–59	1535	28.91
60–69	504	9.49

### 2.1 Development of Preliminary Scales

First, the location and the clinical sign for each type of wrinkle were clearly defined. The photos were then categorized based on the wrinkles' severity level: mild, moderate, or severe. A set of photos most representative of mild and moderate severity was selected to serve as anchor points. Anchor points were graded separately and were aligned across the graders in different regions of China in SGS before confirmation. The photos selected as anchors had to meet the following requirements: Targeted wrinkles having distinct contrasts that were not impacted by gloss (e.g., from excessive sebum), facial or scalp hair not covering the area of interest, no scarring or significant dyspigmentation that may influence the quality of scales (e.g., the observation of wrinkles), etc. In addition, the photos selected should have clear resolution. The cropped, zoomed‐in photos and fullface photos were saved. Thereafter, photos of increased severity were added according to a stepwise approach.

### 2.2 Statistical Method

Intraclass correlation coefficient (ICC) by SPSS 28.0 was used in all the validation tests as summarized in Table 2.

**TABLE 2:** ICC interpretation and its application in different validation tests.

Validation test type	ICC type^Koo and Li^ [6]	Interpretation	Criteria [6]
Intra‐grader repeatability	ICC (2,1)	Two‐way random (both graders and subjects were considered as random effect), and the absolute agreement across different time points (different trials) for multi‐graders were tested	– ICC < 0.5 poor reliability – 0.5 < ICC < 0.75 moderate reliability – 0.75 < ICC < 0.9 good reliability – ICC > 0.9 excellent reliability
Inter‐grader repeatability	ICC (3,1)	Two‐way mixed model, multiple‐graders measurements' consistency was tested	
Ease to use	ICC (3,1)	Two‐way mixed model, multiple‐graders measurements' consistency was tested	

*Note:* All the ICC values were calculated based on 95% confidence intervals.

### 2.3 Intra‐Grader Repeatability

Five graders, from each SGS testing center including Guangzhou (GZ), Shanghai (SH), Hangzhou (HZ), Xiamen (XM), and Qingdao (QD), participated in the validation. Separate sets of 20 photos with a full range of severities were selected for each of the following: crow's feet wrinkle, glabellar wrinkle, forehead wrinkle, and nasolabial folds. All graders were asked to grade the photos independently on a standard screen (EIZO, ColorEdge CG2700S, 2560*1440 for resolution) using the exact same settings and the same distance to the screen (40–60 cm). Each grader performed the grading at day 0 (D0) and again after 1 week with the sequence of the photos randomized. Then the Intraclass Correlation Coefficient, ICC (2,1) was calculated to test the repeatability for each grader across different time points. This is because the graders are considered a random sample from a large population of potential graders, and the results can be generalized to other graders.

### 2.4 Inter‐Grader Reliability

Based on the above grading data, ICC (3,1) was calculated to analyze the reliability across different graders for all the 4 types of wrinkles. ICC (3,1) is intended to assess the reliability of measurements made by a fixed set of graders where the photos (subjects) are randomly selected.

### 2.5 Ease of Use

The usability test further established the robustness of the scale by assessing how less trained graders or untrained naïve consumers can grade wrinkles using this set of scales. This test also demonstrates the ease of training new individuals to use the scales for grading. Analysis of data consistency between the naïve graders and a trained expert grader was conducted. Three naïve graders (2 female and 1 male) without any experience in the cosmetics area were recruited to evaluate the facial wrinkles of the photo sets used above. Hard copies of the photo‐numerical scales were distributed to them as a reference for grading. Then ICC (3,1) was employed to analyze the correlation between naïve graders and the trained expert grader from SH site.

### 2.6 Validation by Primos (Objective Measurement)

3D imaging of facial wrinkles was captured using Primos‐CR (Canfield Scientific, Fairfield, NJ) in parallel with VISIA‐CR 2D image capture for a small set of clinical study participants (*n* = 47). Evaluation of facial wrinkles was conducted using the previously established photo scales. Primos images were analyzed for wrinkle depth and volume at defined areas using Canfield's analysis software. Correlation analysis was run to examine the relationship between the Primos results and clinical evaluation scores.

### 2.7 Evaluating Suitability Compared to Caucasian Skin or Mixed Population Skin Scales

A new set of Chinese photos (*n* = 20, including two sets of duplicates as described below) was selected to compare the grading difference by using both the Caucasian/mixed population‐based and Chinese scales. The Caucasian/mixed population skin scales were previously established by the SGS American team (unpublished data). Forehead, glabellar, and crow's feet wrinkles were assessed via the Caucasian‐only skin scale, while the nasolabial fold was evaluated by using a mixed population scale. The randomly selected photos reflected a wide range of wrinkle severities. Each grader performed grading based on the corresponding scale—Caucasian/mixed population or Chinese—and they were performed 1 week apart. To increase the robustness of this experiment, two sets of duplicate photos were randomly inserted into the photo set, which was blinded to the graders. By comparing the duplicate photos, the graders' sensitivity and consistency were assessed. Descriptive statistics were conducted to generate mean, standard deviation, minimum and maximum, and data distribution between the two scales represented via a Box plot. Since the data was ranking scores under two related pairs, the Wilcoxon signed‐rank test was used to do statistical analysis. The null hypothesis for it is that the grading results based on Chinese population scales are equal to the ones based on Caucasian or mixed population scales.

## 3 Results

### 3.1 Establishment of Photo‐Numeric Scales

**FIGURE 1:**
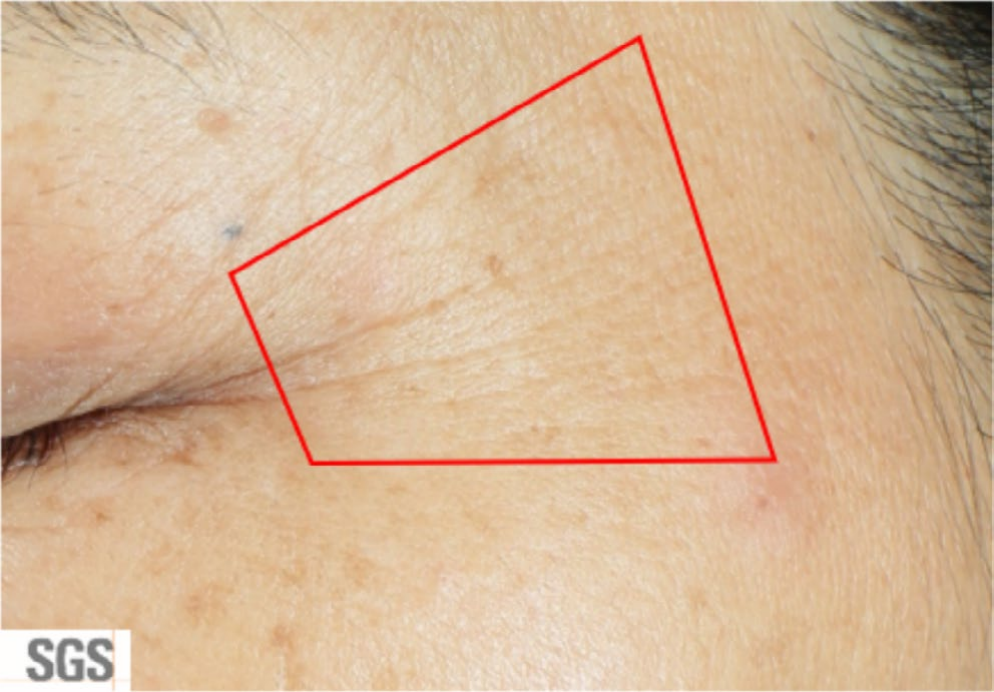
The definition of Crow's feet wrinkles. Crow's feet wrinkles are wrinkles at the area of the outer eye corner starting from about 5 mm away from the corner of the eye. Laterally fan‐shaped area extending from the outer corner of the eye toward the hairline, excluding the part where upper eyelid droops. Grading of crow's feet winkles considers the depth, number and length of wrinkles. This definition is also considered wrinkles at the temple area.

**FIGURE 2:**
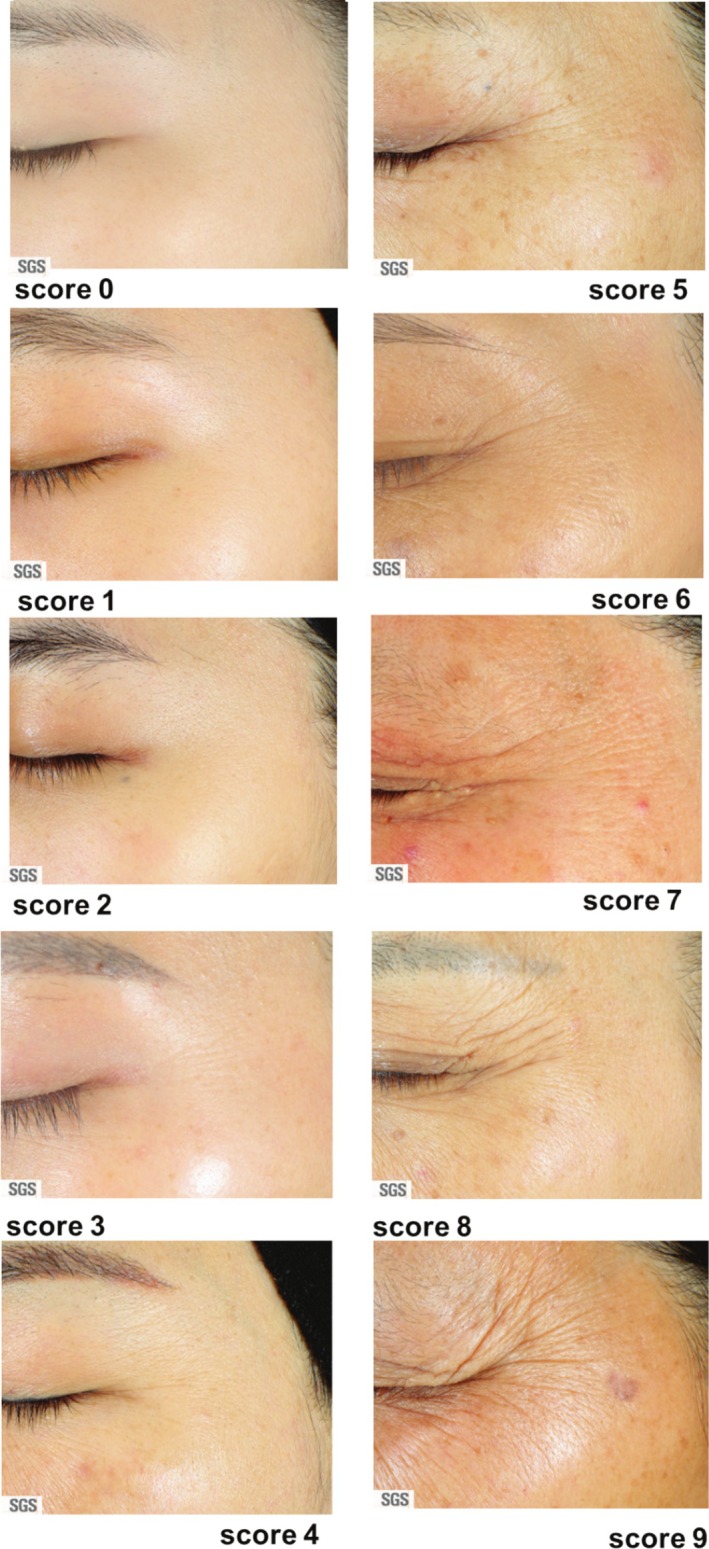
Representative images for 0–9 Photo‐numerical scales for crow's feet wrinkles.

This new set of photo‐numeric scales for facial wrinkles was developed according to the basic principles proposed by Griffiths et al. in 1992 [7]. The 10‐point 0–9 scales cover the range of mild, moderate, and severe conditions with further distinction within each condition. For each specific region, the location of each wrinkle area was clearly defined (Figure 1 and Appendix S1). The considerations of wrinkle scores include the depth, number, and length of wrinkles within each defined area. A detailed description for each score is listed in Table 3 below. An example of the photo scale for crow's feet wrinkles is shown in Figure 2, and the representative images for the other facial locations, forehead, glabella, and nasolabial were shown in the Appendix S1 (Figures S1–S3).

**TABLE 3:** Description of each score point for Crow's feet wrinkle.

	Score	Description
Mild	0	Smooth, no wrinkles
	1	Smooth, minimal, very slight, almost invisible wrinkles
	2	Almost smooth, slight and very superficial wrinkles
	3	Short and superficial wrinkles
Moderate	4	Well defined wrinkles with clear boundary, but not deep
	5	Well defined wrinkles with clear boundary, at least one wrinkle is deep
	6	Rough texture, moderately deep wrinkles
Severe	7	Rough texture, deep and long wrinkles, but the amount is not remarkable
	8	Rough texture, many deep and long wrinkles
	9	Numerous, deep, and long wrinkles

### 3.2 Validation of the Photo‐Numeric Scales

This set of photo‐numeric scales of facial wrinkles was validated for its repeatability, reproducibility, and usability, three essential criteria for an assessment scale.

#### 3.2.1 Repeatability Assessed by Intra‐Grader Validation

Repeatability is one of the most important prerequisites in setting a standard. This was assessed by evaluating the grading consistency of a single grader across different time points.

Five graders, each from a different clinical site, participated in this exercise. Each grader graded a set of photos according to the newly established photo‐numeric scales twice, 1 week apart. ICC (2,1) was calculated to evaluate the intra‐grader agreement, consistency of each grader with themselves. All the values were > 0.9 with *p* < 0.001, indicating that each grader's grading scores were very consistent and repeatable (excellent reliability).

#### 3.2.2 Reproducibility Assessed by Inter‐Grader Validation

Reproducibility is another crucial prerequisite for one method to become a standard. Reproducibility can justify longitudinal studies design, or the comparability of multicell studies performed at different locations. This was assessed by analyzing the data across different graders.

Based on the same data generated by the above intra‐grader repeatability, ICC (3,1) was employed to test the inter‐grader reproducibility. For all the parameters, ICC was > 0.8 with *p* < 0.001, indicating that the grading scores were very consistent among different graders.

#### 3.2.3 Usability Assessed by Naive Grader Grading

Compared to the expert grader, scores from the naïve graders showed very good alignment with that of the expert grader for wrinkles in the glabella, forehead, and crow's feet regions and to lesser extent in the nasolabial fold region (Table 6).

#### 3.2.4 Validation of the Photo Scales With Instrumental Data

To assess if the photo atlas correlates with objective instrumental data, we identified two projects in which both clinical grading and Primos data were captured simultaneously. Primos^CR^ used a fringe projection system to measure wrinkle depth and volume, two key aspects of wrinkle severity grading.

**FIGURE 3:**
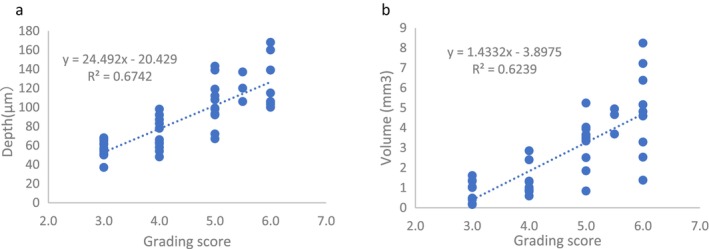
Correlation of Primos wrinkle analysis data and grading data for glabellar wrinkles. (a) The correlation of depth results and grading score; (b) The correlation of volume results and grading score.

The subset of data from two studies were combined and analyzed, only the grading score range from 3 to 6 being included, a core score range targeted by the cosmetic industry. As shown in Figure 3a,b, the grading score showed a high linear correlation for both wrinkle depth and volume results with *r* ^2^ = 0.67 and 0.62, respectively.

### 3.3 Comparison of Chinese‐Specific Atlas to Caucasian/Mixed Population Skin Scales

**FIGURE 4:**
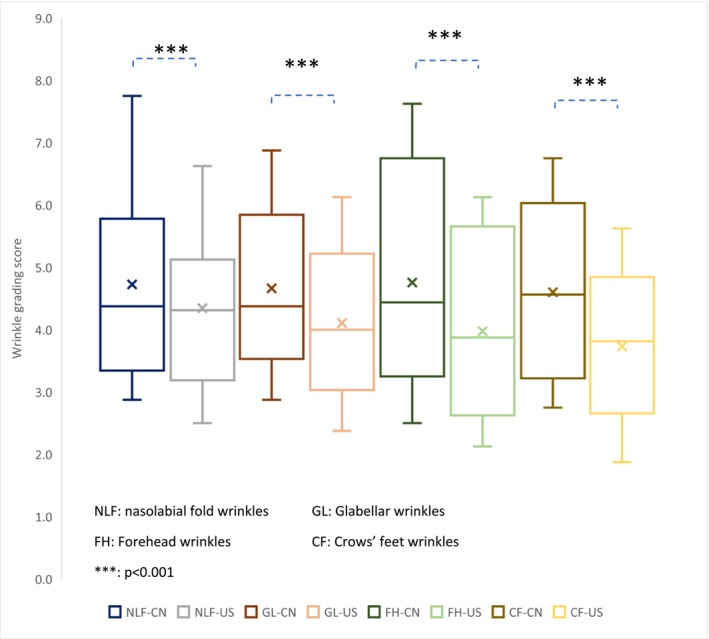
Data distribution comparison between Caucasian skin scales and Chinese ones.

The differences in compatibility between the Chinese‐specific scales and previously established Caucasian/mixed population scales were compared as shown with Box Plot in Figure 4. For glabellar, forehead, and crow's feet wrinkles, the median values by the Chinese scale were higher than the ones by the Caucasian scale, and the mean values of all four wrinkles with Chinese scales were significantly higher than Caucasian scales (*p* < 0.001, detailed data not shown). For nasolabial fold wrinkles, the median values were very close, which is mainly because Chinese panelists tend to have large fat pads on the upper cheek area and the soft tissue descent caused by gravity might contribute to the appearance [1]. Additionally, the comparison with the mixed population scale also mitigated the difference. Moreover, the data range of Chinese skin scales appears wider, especially for forehead wrinkles, indicating that the new scales' alignment and high compatibility with the Chinese subjects' wrinkle severities.

In terms of the grading of duplicate sets of photos, the results for the graders from GZ, HZ, XM, and SH showed 100% equivalent, while the results of the grader from HZ showed half‐point variation for one set of duplicate photos when evaluating crow's feet wrinkles using Caucasian‐specific scales.

## 4 Discussion

Research has demonstrated that wrinkle onset is delayed by 10 years in Chinese women compared with French women [5]. Consequently, it is expected that the range of wrinkle severity in the Chinese population will be different from the Caucasian population. It is crucial to have a comprehensive scale that can be used to evaluate the performance of cosmetics. Despite the known differences between ethnicities, most published scales have been developed based on a Caucasian population [8, 9, 10, 11, 12, 13, 14, 15, 16, 17]. Moreover, these scales were developed to demonstrate the effect of plastic surgery, aesthetic medicine (botulinum toxins injections, dermal fillers, or laser treatment) or pharmaceutical (retinoid drugs) interventions on wrinkle severity often beyond those addressed by cosmetics. Some Asian skin atlases have also been published by Roland B [18], however, the scale length across different skin attributes differs, and other Asian publications are limited to a 5‐point scale [19]. For each skin attribute in previously established Asian skin atlases, the severity change along the scale varies substantially. In other words, the step increment is smaller for the lower score side, while it is larger for the higher score side.

Forehead, glabellar, crow's feet, and nasolabial fold wrinkle scales were established based on the Griffith principle. The 10‐point scale was defined harmoniously, where score 0–3 is categorized as mild level, 4–6 is moderate level, and 7–9 is considered a severe level. This harmonizing principle makes the scales very easy to master by graders. By contrast, previously established scales of different length across varying skin attributes can lead to confusion because the same score value may reflect different severity levels for different parameters. Additionally, the 10‐point scale with more detailed and refined points is more relevant to the cosmetic industry compared to a 5‐point score in clinical aesthetic medicine since the latter can have a more dramatic effect compared to cosmetic formulation treatment. The 10‐point scale was designed to also increase the sensitivity of the scales.

The Chinese‐specific facial wrinkle scales were subjected to rigorous validation to demonstrate their repeatability, reliability, and ease‐of‐use. Inter‐ and intra‐grader validation data among 5 graders from different regions in China showed very high correlation scores with ICC > 0.9 for intra‐grader and ICC > 0.8 for inter‐grader validation (Tables 4 and 5). The results indicate that the scales are highly reproducible and reliable when used by trained graders. These scales will enable comparability in longitudinal studies and multisite studies with well‐trained graders. When naïve graders were asked to grade wrinkle conditions using the photo atlas as a reference, all 3 were able to grade with ease, and their scores showed robust alignment with scores from an expert grader, indicating that the scales can facilitate the training of new graders.

**TABLE 4:** Intra‐grader repeatability (ICC (2,1)).

	Nasolabial folds	Glabellar wrinkles	Forehead wrinkles	Crow's feet wrinkles
Grader—GZ	0.991	0.987	0.995	0.997
Grader—HZ	0.964	0.980	0.962	0.922
Grader—XM	0.988	0.998	0.997	0.994
Grader—QD	0.942	0.966	0.972	0.937
Grader—SH	0.975	0.997	0.996	0.995

**TABLE 5:** Inter‐grader reliability (ICC (3,1)).

	1st round	2nd round
Nasolabial fold	0.862	0.850
Glabellar wrinkle	0.951	0.964
Forehead winkle	0.947	0.960
Crow's feet wrinkle	0.933	0.964

Photos used to generate these scales were collected from multiple clinical sites in different regions of China, from east (SH) to south (GZ). It covers locations of varying climate (dry or humid, cold or hot) and of varying lifestyles (stress levels, pollution levels). The scales were validated and used across these regions as well, which suggests that the scales are generally applicable to their local populations. In other words, the climate and lifestyle differences contribute to the aging process, although the harsher climate and more stressful lifestyle could make skin aging faster, our scale severity range can cover the potential regional difference. While inland arid or high altitude regions with greater UV irradiation are not covered, whether extreme climate conditions could push the wrinkle severity to a higher level needs to be investigated further in the future.

The scales were further validated against objective instrumental data generated from Primos^CR^ analysis. As shown in Figure 3a,b, the comparison between grading scores and Primos data showed a strong correlation between wrinkles' volume and grading scores (*r* = 0.79), and between wrinkles' depth and grading scores as well (*r* = 0.82). This crucial validation revealed that the core region of our scales (mild to moderate, which is the general target population for cosmetic products) has a linear stepwise increment. While this also unfolded the limitation of our research, more work is needed in the future to run correlation for very mild (score 0–2) and severe (score 7–9) wrinkles.

When comparing to previously used Caucasian skin scales, grading scores obtained from the Chinese wrinkle scales presented significantly higher mean wrinkle severity values (*p* < 0.001) for the same set of photos and a wider data range (Figure 4) including forehead, glabellar, and crow's feet wrinkles. The higher mean value for wrinkle scores, indicating a score of higher severity, is consistent with the fact that wrinkle onset in the Chinese population is delayed compared to Caucasians. For nasolabial fold wrinkles, which were based on a mixed population, the mean value was also statistically higher, while the median value was closer. The wider data range suggests that the Chinese wrinkle scale may better detect the subtle differences of certain wrinkle conditions, making it potentially more sensitive when used in Chinese populations. Together, the new scales are scientifically sound, practical, and suitable for a Chinese population.

When utilizing the wrinkle grading scales, a half‐point is allowable; however, no half‐point photos are included in the photo scale. This is intended to give more flexibility when visible improvement is evident, but a full point change is yet to be reached. The linearity of the scale makes it easier to extrapolate the half‐point condition. Allowing half‐point grading can potentially increase the sensitivity of clinical grading and allow differentiation of the performance of two different skincare products. It is, however, expected that the efficacy of a product measured using these scales or the ones developed with Caucasian or mixed populations will not be substantially different.

Although the presented scales are easy to use (Table 6), intensive and continuous training of graders is still of utmost importance. Wrinkle patterns have many different presentations among different individuals and populations. For example, crow's feet wrinkles can be full‐fan, lower‐fan, upper‐fan, and central‐fan [20]. The photo‐numerical scales were designed to be representative of the wrinkle's severity at each score level and to show progression of the severity. It is not possible to include all the varying patterns of each wrinkle type. It is only with extensive training and experience that a grader can truly master the skill of clinical grading, and the photo‐numeric scales provide invaluable guidance in this process.

**TABLE 6:** Agreement between the grading of Naïve graders and the professional grader in SH (ICC (2,1)).

	Nasolabial fold	Glabellar wrinkles	Forehead wrinkles	Crow's feet wrinkles
Naïve grader 1	0.742	0.915	0.897	0.941
Naïve grader 2	0.789	0.855	0.878	0.935
Naïve grader 3	0.712	0.954	0.897	0.925

In conclusion, the photo‐numeric wrinkle scales have demonstrated robust repeatability and reproducibility, strong practicality, and reliable correlation with objective measurement. Given the tremendous sample size covering various regions in China, these scales are beneficial tools to evaluate the effectiveness of cosmetic products in a Chinese population.

Only limited types of wrinkles were reported here. To cover a wholistic range of skin aging signs, other types of wrinkles and lines scales, together with skin tone associated attributes, are under development to fill the gap of current work. Regarding the difference in the pattern evolving for each type of wrinkle, it is quite challenging to define. For instance, glabellar or forehead wrinkles are often influenced by habitual facial expressions, whereas nasolabial folds and marionette lines are more closely tied to facial architecture. More knowledge and understanding are being built in this area to further unfold the skin aging process.

## Author Contributions

Xiaomin Zhao is the project initiator, she co‐worked with Lily I. Jiang on ideation, the implementation plan to establish Chinese population scale, developing wholistic validation plan. Lily I. Jiang and Yang Yang led the implementation of the project; Xiaomin Zhao checked in and discussed together the reasonability and representativeness of each photo regularly. All of them worked together on data analysis. Lily I. Jiang and Xiaomin Zhao contributed equally to manuscript drafting. Hao Ouyang gave some important guidance on data analysis, and how to compare with Caucasian skin or mixed population scale. Yan Zhong and Claude Saliou intensively reviewed the article and advised some critical comments in the structure of the article, and many details.

## Conflicts of Interest

The authors declare no conflicts of interest.

## Supporting information


**Appendix S1:** jocd70451‐sup‐0001‐AppendixS1.docx.

## Data Availability

The data that support the findings of this study are available on request from the corresponding author. The data are not publicly available due to privacy or ethical restrictions.
