# Supplementary material for: Development and Validation of Photo‐Numerical Scales for Facial Wrinkles on Chinese Population
Source: J Cosmet Dermatol. 2025 Sep 18;24(9):e70451. doi: 10.1111/jocd.70451 (PMC12445401; doi:10.1111/jocd.70451)
Supplement: Supplementary file 1 — Appendix S1: jocd70451‐sup‐0001‐AppendixS1.docx. [file JOCD-24-e70451-s001.docx]

Appendix S1 or Supplemental Information

**Ethical statement**

The study involved 5,310 Chinese females aged 18 to 69 years which was collected through multiple clinical studies over 2.5 years from Jan 2021 to Aug 2023 utilizing Visia CR image capturing system. All clinical studies involved to collect photos were conducted following Good Clinical Practice guidelines and China’s regulations. Written informed consent and photo release were properly obtained for each subject.


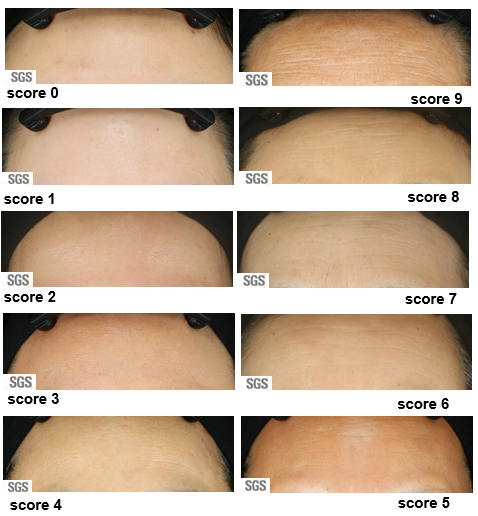


**Figure S1 Photo-numerical scales for forehead wrinkles**


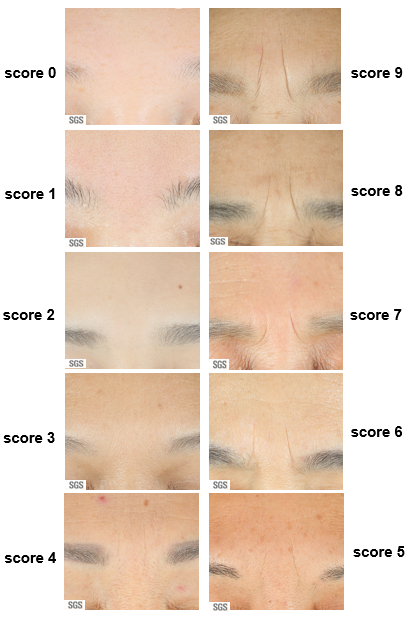


**Figure S2 Photo-numerical scales for glabellar wrinkles**


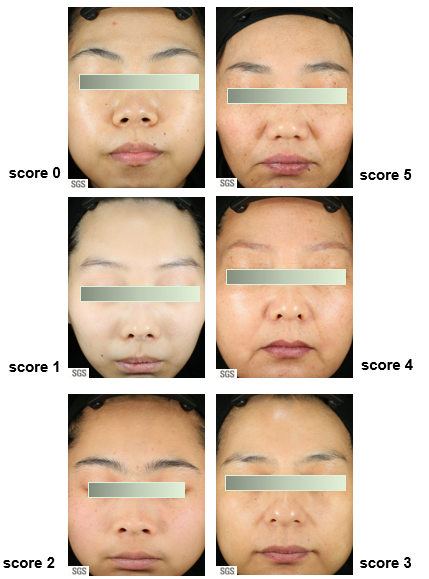


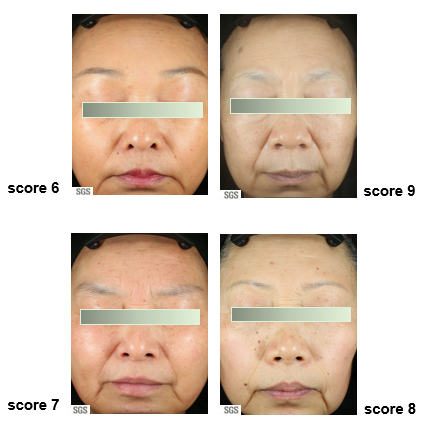


**Figure S3 Photo-numerical scales for nasolabial fold wrinkles**
